# Truth or lie: Exploring the language of deception

**DOI:** 10.1371/journal.pone.0281179

**Published:** 2023-02-02

**Authors:** Justyna Sarzynska-Wawer, Aleksandra Pawlak, Julia Szymanowska, Krzysztof Hanusz, Aleksander Wawer

**Affiliations:** 1 Institute of Psychology, Polish Academy of Sciences, Warszawa, Poland; 2 Polish-Japanese Academy of Information Technology, Warszawa, Poland; 3 University of Social Sciences and Humanities, Warszawa, Poland; 4 Institute of Computer Sciences, Polish Academy of Sciences, Warszawa, Poland; University of Padova, ITALY

## Abstract

Lying appears in everyday oral and written communication. As a consequence, detecting it on the basis of linguistic analysis is particularly important. Our study aimed to verify whether the differences between true and false statements in terms of complexity and sentiment that were reported in previous studies can be confirmed using tools dedicated to measuring those factors. Further, we investigated whether linguistic features that differentiate true and false utterances in English—namely utterance length, concreteness, and particular parts-of-speech—are also present in the Polish language. We analyzed nearly 1,500 true and false statements, half of which were transcripts while the other half were written statements. Our results show that false statements are less complex in terms of vocabulary, are more concise and concrete, and have more positive words and fewer negative words. We found no significant differences between spoken and written lies. Using this data, we built classifiers to automatically distinguish true from false utterances, achieving an accuracy of 60%. Our results provide a significant contribution to previous conclusions regarding linguistic deception indicators.

## Introduction

Lying is a part of everyday human communication, and most of us engage in it—studies show that people tell an average of one to two lies per day [1]. It has been reported that lies are also increasingly common in computer-mediated communication and in text-based interactions [2]. As a consequence, their detection through language analysis is critical in contexts that rely on truthful inputs.

To date, many studies have attempted to capture the differences between true and false statements. These differences may be related to specific types of emotions experienced by liars, cognitive processes occurring while lying, and self-presentation strategies to control behavior by liars [3]. According to the emotional approach, lying can trigger emotions such as excitement, fear, and guilt [4]. They can influence the behavior of a liar and how they speak, e.g., by increasing the use of words with emotional tones or negations [5]. The cognitive approach emphasizes that lying is more cognitively demanding than telling the truth. It requires the involvement of working memory, cognitive control, and shifting attention [6]. This cognitive load may be visible in how a liar speaks (e.g., slower speech pace, mistakes) and also in how they construct false statements, which may be simpler and shorter than true statements. Finally, according to the self-presentation approach proposed by DePaulo et al. [7], liars are less direct than truth-tellers out of concern for their own image; they distance themselves more from the lies they utter and provide fewer details.

These theoretical assumptions have been confirmed in numerous studies, including a meta-analysis conducted by Hauch et al. [8] in which written and transcribed statements from 44 different studies were analyzed. The authors of studies included in the meta-analysis used a variety of software to identify and quantify linguistic cues to deception. The English version of the Linguistic Inquiry and Word Count (LIWC) [9] was the most common. LIWC analyzes transcripts with a dictionary-based approach in which each word is compared against a file of words divided into 74 linguistic dimensions. After counting the number of words in each category, the output is given as a percentage of the total words in the text sample. The results showed that liars express more negative emotion words, distance themselves from events by using fewer self-references (first-person pronouns) and more other-references (second and third-person pronouns), and—as predicted by the cognitive load approach—constructed shorter (fewer words and fewer sentences), less elaborated (fewer different words) and less complex (fewer exclusive words) stories. Some studies included in Hauch’s meta-analysis [8] suggested that liars may also use more negations and over-generalizations. False statements are also less abstract. Newman et al. [10] suggest that due to cognitive load, liars may focus more on simple, concrete verbs rather than evaluations and judgments because the former are more accessible and easier to combine into a false narrative.

Interestingly, different results were obtained in the few studies that analyzed non-English language statements. Schelleman-Offermans and Mercklerbach [11] analyzed statements in Dutch. They failed to confirm differences in complexity between true and false statements as measured by the use of motion verbs. They also found no differences in the use of emotionally negative terms or self-references. False statements differed from true ones only in their less frequent use of exclusive words. Research conducted by Masip et al. [12] on Spanish statements also yielded different results than those repeated in English: true statements differed from false ones only in the use of prepositions (there were more of them in true statements than in false ones) and words related to positive emotions, positive feelings, affective processes, and achievements, which were less common in false statements.

Accordingly, the main goal of our research was to identify differences between true and false statements in the Polish language. Polish is a Slavic language in which grammatical categories are implemented through word inflection, thanks to a rich morphology. This, in turn, allows for a relative freedom in word order and may affect the complexity of statements. Moreover, the Polish language is characterized by the use of reflexive pronouns, which indicate that an action is directed at a performer. Correlations of various part-of-speech classes to either truth or lies have been reported for the English language, with ‘I’ words (self-reference) being found as most relevant to detecting lies. In contrast to English, in Polish self-reference is expressed in either morphology or in part by reflexive pronouns. In this article, we focus on the Polish language and investigate possible differences in the distribution of these properties between true and false statements using automated tools such as part-of-speech taggers and dependency parsers.

In testing the differences between true and false statements, we tried wherever possible to replace dictionary methods with non-dictionary methods and general tools with specific tools tailored to a particular variable. We wanted to move away from dictionary methods primarily because they are developed for a specific language and often lack equivalents for others. We therefore tried to propose methods that can be used independently of language and that are freely available. Where this was not possible, we have chosen dictionary tools developed for a specific variable as they provide better recall and, therefore, are more accurate than general dictionaries.

Below, we describe which differences between true and false statements were analyzed in our research and with which tools. We also state a hypothesis for each variable based on the results of previous research. Hypotheses are posed separately for written and transcribed statements because previous research has shown some differences depending on the type of statement. In writing, liars have more time to plan their utterances and can edit them. In some studies where only written statements were analyzed, this affected—among other things—their length: subjects used more words when lying than when they were telling the truth [13].

### Cognitive load approach

#### Complexity

Cognitive load may reduce the complexity of false statements, affecting the simplification of both vocabulary and syntax. Therefore, we decided to use three different measures: Mean Dependency Distance (MDD) and Mean Hierarchical Distance (MHD), which are related to syntax, and Gunning Fog Index (FOG), which is related to vocabulary. All three measures are described in detail in the “Method” section.

In general, the differences in sentence characteristics generated under different cognitive loads can be measured by dedicated indexes that are sensitive to changes in load. One such tool is dependency distance—proposed by Heringer [14]—defined as the number of words intervening between two syntactically related words. Dependency distance can measure the memory burden imposed on language processing and reflects the dynamic cognitive load of language generation under various conditions [15]. Using automated dependency parsing, measures such as MDD and MHD offer two different implementations of the concept of dependency distance.

The third measure, namely the Gunning Fog Index (FOG), takes a different approach to estimating text complexity: it computes ratios of complex words in terms of the number of syllables to all words. The FOG is an automated readability index, which translates a text into the number of years of formal education a person needs to understand it the first time they read it. FOG is usually used to confirm that a text can easily be read by the intended audience and has also been used for cognitive load measurements. In deception detection research, it was used by Pérez et al. [16].

MDD and MHD have not yet been used in deception research; we believe that their use in combination with FOG could help measure complexity through readability and syntax, and more accurately determine the nature of complexity differences. Regardless of the type of statement (written/transcribed), we postulate that true statements will be more complex than false statements in terms of all indicators.

#### Length

The length of a text may also indicate its complexity, and we used three variables to measure it: the number of sentences, the number of characters used in the statement, and the number of tokens. According to Manning et al. [17], a token is an instance of a sequence of characters in some particular document that are grouped together as a functional semantic unit for processing. Tokens, usually corresponding to words, are the result of splitting an input text into pieces and sometimes discarding certain characters, such as punctuation. These measures have been used in research on lying many times and in several different languages. We assume that in the case of transcribed statements, true statements will be longer than false statements. In the case of written statements, the opposite will be true: false statements will be longer. The differences will be visible for all measures.

#### Concreteness

To inspect differences in the concreteness of true and false statements, we decided to use the Linguistic Category Model (LCM) proposed by Semin and Fiedler [18]. This model is based on dividing words into categories reflecting their abstractness level. The authors distinguished four categories, including three types of verbs and adjectives: Descriptive Action Verbs (DAVs), Interpretative Action Verbs (IAVs), State Verbs (SVs), and Adjectives (ADJs). The most abstract category in LCM is adjectives (ADJ), such as “good” or “smart”, which are used to describe highly conceptual dispositions and personality traits. Adjectives enable generalizations across situations, objects, or specific behavioral events.

The most abstract type of verbs are state verbs (SVs), which are mainly used to describe emotional or mental states (e.g., “love”, “think”). They lack a clear beginning and end and make no direct references to a specific behavioral episode or situation, although they do refer to a particular object. The second type of verb is the interpretative action verb (IAV). IAVs describe a general class of behaviors without identifying the specific behavior they refer to in a given context (e.g., “help”). The last and most specific type of verbs are descriptive action verbs (DAV). DAVs are verbs that describe a single and observable event defined by at least one physically invariant feature and with a clear beginning and end (e.g., “call”).

According to Semin and Fiedler [18], differences in the level of language abstractness can be observed even when people describe the same events. The authors claim that due to the development of the LCM itself, we can evaluate the degree to which people reveal their abstract or concrete thoughts in language usage. LCM has so far been used to study the abstractness of deceptive statements. Promising results were obtained by Louwerse et al. [19], who used this tool to predict whether an email consists of fraudulent information.

Again, we believe that this tool potentially provides the most accurate estimate of concreteness, as it covers a much larger number of words than the individual word classes in LIWC or other general dictionaries previously used for this purpose. We assume that true statements, whether written or transcribed, will be more abstract than false ones. The key for us will be the overall abstractness score; we have no directional hypotheses about the occurrence of particular categories of verbs and adjectives.

### Emotional approach

#### Sentiment and negations

In order to study the use of emotionally charged words by liars, we also decided to choose a different method than LIWC and used sentiment analysis. Sentiment analysis is a technique that uses natural language processing to evaluate a text’s emotional emphasis. The algorithms used in sentiment analysis allow for categorizing expressions/text parts as neutral, positive, or negative. Sentiment analysis is often used in research on reviews, opinions and attitudes, and often consists of counting word occurrences with a positive or negative meaning, such as “beautiful” or “horrible.”

Machine learning and deep learning are most often used for this purpose and achieve high accuracy. Unfortunately, they are usually limited to a specific topic or type of text (domain-dependent). Therefore, in our research, we decided to use a method based on an open-domain sentiment dictionary. Unlike the domain-dependent type, it is more universal and does not categorize entire texts. However, it does allow for an accurate count of positive and negative words appearing in the text.

Sentiment analysis allows us to categorize many more words than is possible with LIWC, making our measurement more accurate. In contrast, we measured the prevalence of negation as has been done in other studies—using a list of words (such as “no” or “never”) and counting the occurrences of these words in the statements. In both written and transcribed sentences, we expect lies to use more negations and words denoting negative emotions and fewer positive emotions.

### Self-presentation approach

#### Part-of-speech and over-generalizations

We tested whether liars distance themselves from their lies by using fewer first-person pronouns and more third-person pronouns. Since previous studies have found differences in other parts of speech (e.g., participles) and such studies are lacking in Polish, we decided to analyze the frequency of occurrence of many other parts of speech (see S1 File for a complete list). We decided to treat the analysis of parts of speech in an exploratory manner and only hypothesized about pronoun use. Namely, we assume that the differences in the frequency of using first and third-person pronouns shown in English will not occur in Polish, where the personal pronoun is often omitted and the person is included in the form of a verb. These same assumptions apply to written and transcribed statements.

Another way to distance oneself from lies can be to use over-generalizations. We measured them by comparing words from statements with lists of words explicitly created for this variable and by counting the frequency of their occurrence. We hypothesize that there will be more over-generalizations in false written and oral statements than in true statements.

### Summary

To summarize, we aimed to check whether the differences between true and false statements found in English studies would also appear in Polish. We considered variables derived from the underlying models of deception, which have been repeatedly considered as predictive of deception. However, our research used newer and more specialized methods than most previous studies. Our study is the first to use tools dedicated to measuring language syntax complexity and sentiment analysis, and also to compare spoken and written statements since we have true and false statements, both written and transcribed from the same individuals. With this data we built a classifier to automatically distinguish true and false statements.

## Method

### Participants

400 participants aged between 18 and 60 took part in the study (F = 226; M_age_ = 30.58, SD = 9.63). Overall, 4.5 percent of the participants completed primary school, 46.5 percent had a secondary education, and 49 percent had a higher education. Their native language was Polish. Participants were recruited using social media and internet advertising portals. Each participant received 100 PLN (approximately 25 EUR) for taking part in the study. Ethical approval to conduct the study was obtained from the Committee for Ethics of Scientific Research, Institute of Psychology, Polish Academy of Sciences. All subjects gave their written informed consent, and were made aware that they have the right to quit the study at any moment.

### Procedure

In the first step, participants completed a questionnaire about their attitudes towards 12 topics that polarize public opinion. They marked their answers on a Likert scale with response options ranging from -5 “strongly disagree”, 0—“have no opinion”, and 5—“strongly agree.” The experimenter selected the two topics on which each participant marked the most extreme answers (always at least +/-4). Topics included various social, political, economic, and sports issues (for full list see Table 1).

**Table 1:** List of topics: (1) Vaccinations should/should not be compulsory, (2) Polish energy should be based mainly on coal/renewable and non-emission sources, (3) People should/should not eat meat, (4) Smartphones and social media positively/negatively affect interpersonal relationships, (5) Abortion should/should not be legal, (6) God exists/does not exist, (7) Robert Lewandowski is/is not the best Polish football player, (8) Jerzy Zięba’s treatments are/are not effective and help people heal/can harm the sick, (9) Poland should/should not accept more immigrants than today, (10) GM food is/is not safe and useful, and we should/should not invest in these kinds of crops, (11) The political situation in Poland is going in the right/wrong direction, (12) In general, most people can/cannot be trusted, (13) Ewa Chodakowska is/is not the most effective personal trainer in Poland.

	written		transcriptions	
topic	N	%	N	%
1	134	17.6	92	12.5
2	81	10.6	55	7.4
3	76	10	78	10.6
4	54	7.1	55	7.4
5	91	11.9	94	12.7
6	43	5.6	50	6.8
7	60	7.9	53	7.2
8	30	3.9	50	6.8
9	62	8.1	40	5.4
10	34	4.5	34	4.6
11	32	4.2	54	7.3
12	32	4.2	50	6.8
13	30	3.9	31	4.2

The participants were then asked to generate four statements. Two of them were focused on one topic and were expressed orally and recorded. The other two were written (typed) on an online form. One statement on a particular topic was consistent with each participant’s real position, while the other presented an opposing viewpoint. We gathered 1600 statements in total using this method. These included four from each participant: two real statements (one oral and one written) and two false ones (again one oral and one written).

Participants spoke for at least two minutes during their oral statements, and were provided at least five minutes to give written statements. For each statement, the participant’s task was to present their position and to justify it. Participants were encouraged to present both arguments and verifiable facts, as well as their subjective opinions, experiences and feelings. Participants were told that other people would hear their recordings or read their statements and attempt to guess their true views, and that they should therefore be as persuasive and believable as possible while delivering both communications. The statement order, regarding both the type of communication and their truth/falsehood, was randomized.

### Dataset

We analyzed 1498 (760 written statements and 738 transcriptions) out of 1600 statements obtained in the study. 103 statements were excluded from the analysis, including statements made by participants who did not understand the instructions (e.g. they answered truthfully twice or directly admitted to lying), statements that were too short, and written statements consisting of only the sentence equivalents. In the case of some recordings, transcription was not possible or speech was not recorded due to technical problems. An automatic transcription service “Happy Scribe” (https://www.happyscribe.com) was used to transcribe the oral statements. All transcriptions were checked and manually corrected. No modifications were made to the participants’ written statements. Each transcript and each written statement was then saved in a separate text file.

### Analysis techniques

#### Dependency parsing

**Fig 1:**
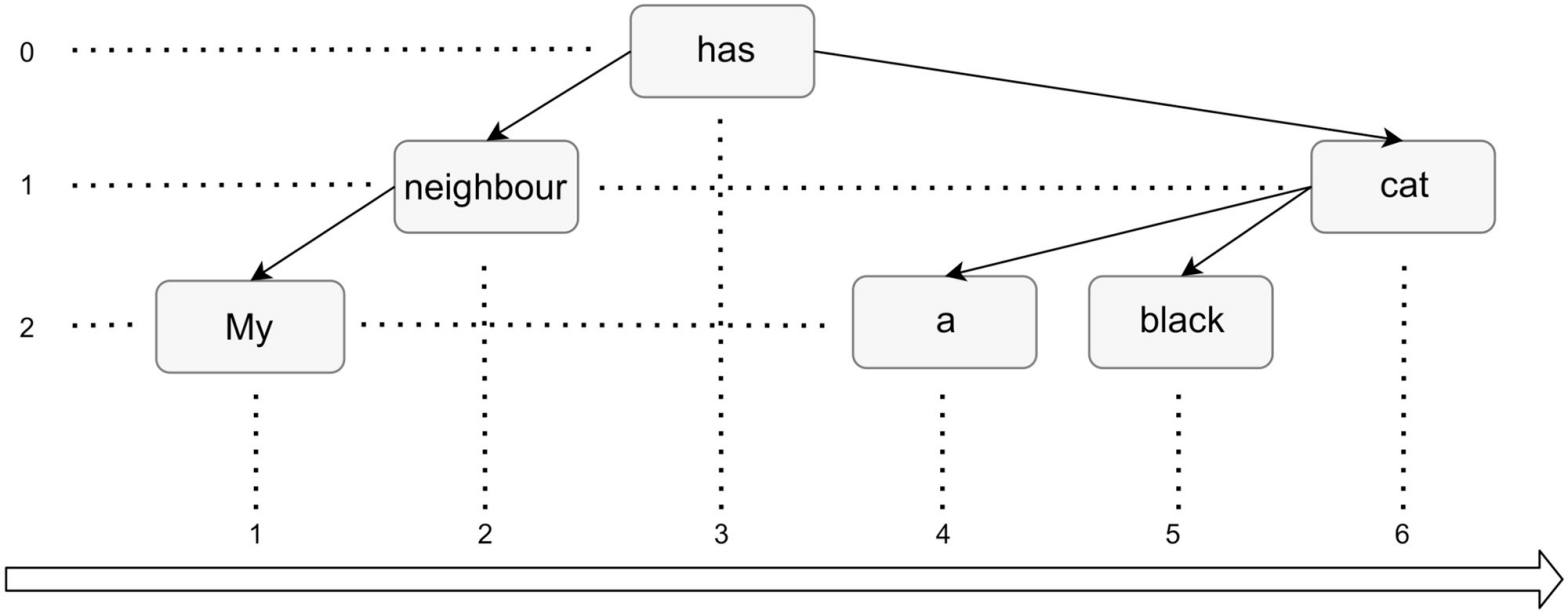
Example dependency tree.

To compute syntactic complexity, we employed two measures that use dependency parsing. This approach to the computerized, automatic syntax analysis of sentences in natural language plays an important role in contemporary speech and language processing systems. In dependency parsing, the syntactic structure of a sentence is described in terms of binary grammatical relations that are defined for pairs of words in a sentence. Relations among the words are directed and labeled, leading from heads to dependents. An example of a dependency parse tree is depicted in Fig 1.

#### Mean Dependency Distance (MDD)

The first measure is mean dependency distance (MDD). It was proposed by Liu [15] as a metric for language comprehension difficulty. MDD uses dependency parsing information and the order of words in a sentence. The basic element of the formula is “dependency distance (DD)”, the word-level (surface) distance between the positions of dependent and head vertices (words).

The averaged DDs for all dependency tree pairs make up the MDD. The formula is defined in Eq 1, where n is the number of dependency pairs in a sentence, and *DD*_*i*_ is the absolute value DD of the i-th dependency distance.

$$\begin{array}{c} MDD=\frac{1}{n}\sum_{i=1}^{n}\left|DD_{i}\right| \end{array}$$ (1)

DD can be positive or negative, as a dependent word and its head word can be located on different sides with respect to each other. Therefore, MDD is the average value of all pairs of absolute dependency distances.

#### Mean Hierarchical Distance (MHD)

The second measure uses only dependency tree distances and does not use word position distances as in MDD. The idea behind the MHD [20] is to utilize the distances between each node and the root. Namely, we take the root of a syntactic tree as a reference point and compute the vertical distance between a node and the root, or the path length on the way from the root to a certain node along the dependency edges. This is defined as “hierarchical distance (HD).” The average value of all hierarchical distances in a sentence is the mean hierarchical distance (MHD), as defined in Eq 2.

$$\begin{array}{c} MHD=\frac{1}{n}\sum_{i=1}^{n}HD_{i} \end{array}$$ (2)

#### Example of MDD and MHD

A dependency parse tree for an input example sentence is presented in Fig 1. We stretched the syntactic structure vertically to better illustrate the concept of hierarchical distances.

Following Eqs 1 and 2, we can calculate the MDD and MHD of the example sentence in Fig 1.

The Mean Dependency Distance is the surface (X-axis in Fig 1) distance between a word and its parent. The word “My” has the word “neighbour” as its dependency parent and the distance between them is 1, as measured on the X-axis. The same situation holds for the words “neighbour” and “has”, hence the distance is 1. The word “black” is between “a” and “cat”, and therefore the distance is 2. The distance from “black” to “cat” is 1. The words “cat” and “has” are two words away (“a” and “black” are between them), hence the distance is 3. The MDD of this sentence is (1 + 1 + 2 + 1 + 3)/5 = 1.6.

The Mean Hierarchical Distance takes into account the vertical distance between each word and the root of the sentence, which is the word “has.” The distance between “my” and “has” is 2 as we move two arrows up, and between “neighbour” and “has” the distance is 1. The distance between “cat” and “has” is also 1. The distance between both “a” and “black” and the word “has” are equal to 2. Therefore, the MHD is computed as (2 + 1 + 1 + 2 + 2)/5 = 1.6.

Contrary to the example shown above, the values for most sentences are usually different. In our study, each sentence from the data set was parsed syntactically using a dependency structure analyzer from the Polish modules of the Spacy tool (https://github.com/ipipan/spacy-pl).

#### Gunning-Fog Index (FOG)

The Gunning Fog Index is a measure of readability. It estimates the years of formal education a person needs to understand a given text upon first reading. Computing this index begins with taking a sample passage of at least 100-words and counting the number of all words, complex words (those containing three or more syllables), and sentences. The formula is illustrated in Eq 3.

$$\begin{array}{c} FOG=0.4[(\frac{words}{sentences})+100(\frac{complex\;words}{words})] \end{array}$$ (3)

We used the implementation from Textstat (https://pypi.org/project/textstat), a Python library to compute readability, complexity, and grade level statistics from text. Textstat supports the Polish language for the calculation of the Gunning Fog Index.

#### Linguistic Category Model (LCM)

In our study, we used the Polish LCM dictionary (LCM-PL; [21]), which contains the 6,000 most frequent Polish verbs. For each participant, the number of tokens (an individual occurrence of a linguistic unit) of a given word type (DAVs, IAVs, SVs, ADJs) in the whole statement was added up. According to the LCM formula, the only type of adjectives that are taken into account are those that modify subject nouns. All the remaining ones are skipped. This information has been determined using the dependency parser from Spacy-PL. We calculated the level of abstraction according to the weighted summation formula recommended by the LCM’s authors (DAV + IAV * 2 + SV * 3 + ADJ * 4). We refer to this formula as the general LCM score.

#### Sentiment

We used a dictionary of 5421 positive and negative words, obtained from two sources. The dictionary was created manually and represents the sentiment of the most frequent sense of a word, and therefore requires no word sense disambiguation. The first source was plWordNet Emo, which contains 32 thousand lexical units manually annotated with sentiment labels on the level of word senses [22]. Sentiment labels were re-assigned and verified to represent the most frequent sense only. The second source is a manually labeled dictionary of 1774 negative and 1493 positive words, pulled from a rule-based sentiment analyzer [23].

#### Part-of-speech

To obtain part-of-speech occurrences, we again used Spacy-PL, which internally uses a bi-LSTM neural network to label the tokens [24]. We used more detailed part-of-speech tags available from the Morfeusz library [25].

## Results

We applied hierarchical regression modeling to analyze differences between truthful and deceitful statements in the context of psycholinguistic quantitative variables related to complexity, length of speech, abstractness, sentiment, and part-of-speech (a full list of variables with descriptive statistics is included in S1 Table). Each model included the same set of three predictors: statement type (true vs. lie), statement form (written vs. transcription), and the interaction of those. The predictor related to the statement form (written vs. transcribed) and the interaction of both predictors acted as control predictors. The following sum contrasts were used: true (-0.5) vs. lie (0.5), and written (-0.5) vs. transcription (0.5).

We took into account the by-subject variability when constructing models with varying intercepts. Models were fitted to data using the R package lme4 [26]. Due to multiple comparisons, the analysis used Holm’s correction. A summary of the models is included in S2 Table. Logarithmic transformation was performed for each dependent variable before visualisation and hierarchical regression modeling. Before the transformation, 1 was added to all observations of each variable to avoid zero values. In the case of variables related to abstractness, sentiment, and part-of-speech, the length of the statement was taken into account: the number of occurrences of a given variable was divided by the number of tokens.

### Complexity

#### MDD, MHD, and FOG

In the case of MDD and MHD, the main effect of statement type (truth/lie) was statistically non-significant, meaning that the tested true statements do not differ from false statements in terms of syntactic complexity. Oral statements were more complex than written statements (MDD:*b* = 0.13, *p* = 0.001; MHD: *b* = 0.12, *p* = 0.001). Further, the results indicate that participants had a higher FOG score for true statements than in the condition of false statements (*b* = -0.059; *p* = 0.004). It was also observed that the FOG score is higher in the transcription condition than in the written statements (*b* = 0.09, *p* = 0.001). No interaction effects were observed for any of the variables.

#### Length

Participants obtained higher results in the condition of true statements than false statements for all of our speech length measures (sentence length: *b* = -0.091, *p* = 0.001; tokens: *b* = -0.184 *p* = 0.001; characters: *b* = -0.188, *p* = 0.001). Oral statements were longer than written statements (sentence length: *b* = -0.31, *p* = 0.001; tokens: *b* = 0.8, *p* = 0.001; characters: *b* = 0.71, *p* = 0.001)). Interaction effects were not observed for any of the variables.

#### Concreteness

No significant differences were found between true and false statements in terms of individual verb categories (DAV, IAV, SV) and adjectives distinguished in the LCM model. In oral statements, the scores for DAV (*b* = 0.09, *p* = 0.001) and SV (*b* = 0.13, *p* = 0.001) were higher than in written statements. The interaction effects were not statistically significant. However, the results indicate that participants obtained higher values in the general LCM score (*b* = -0.12, *p* = 0.013) for true statements than in the condition of false statements. Further, LCM scores (*b* = 0.62, *p* = 0.001) are higher in transcribed statements than in the case of written statements. No interaction effect was observed.

### Sentiment and negations

**Fig 2:**
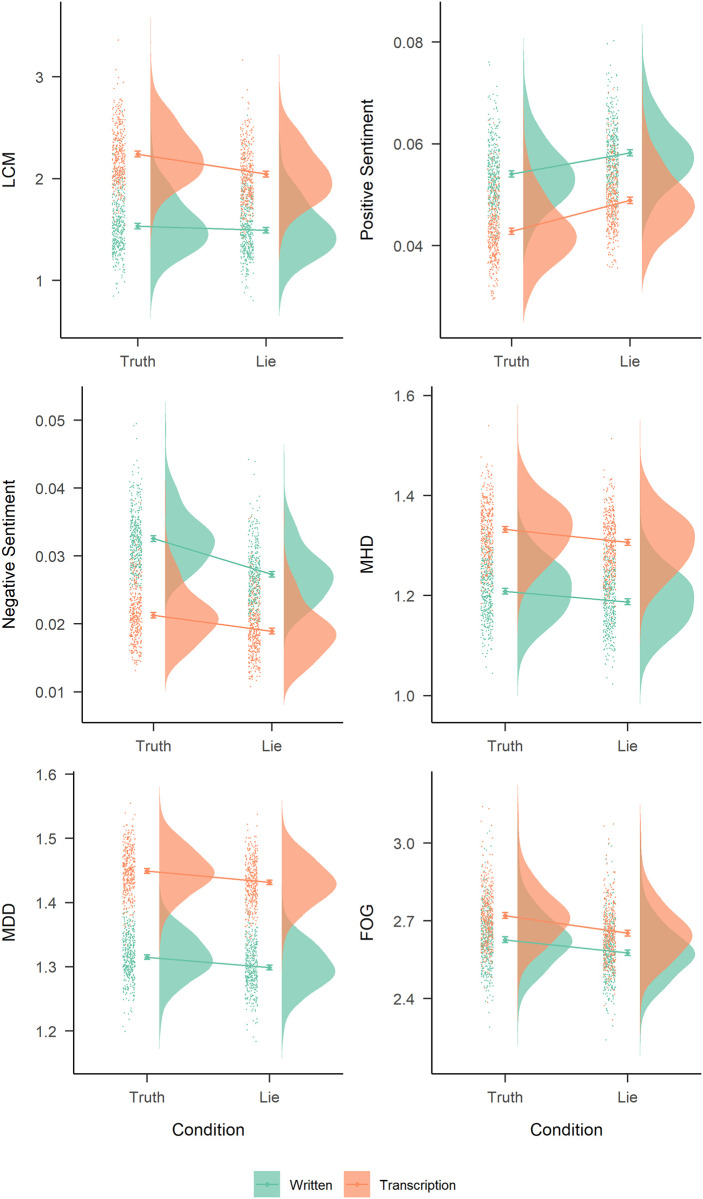
Raincloud plots of the difference between truthful and deceitful responses in predicted values for LCM (general score), positive sentiment, negative sentiment, MHD, MDD, and FOG. Predictions based on the hierarchical models comprised of a log transformed dependent variable and three predictors: truth vs. lie, written vs. transcription, and their interaction.

On one hand, positive sentiment appeared to be higher in the deceitful condition than in the truthful condition (*b* = 0.005, *p* = 0.006), and we also observed that positive sentiment was higher in the written condition (*b* = -0.01, *p* <0.001) than for transcriptions. On the other hand, negative sentiment was higher in the case of false statements than true statements (*b* = -0.004, *p* = 0.006) and in written statements compared to transcripts (*b* = -0.01, *p* <0.001). For both types of sentiment, the interaction of both predictors was not statistically significant. No significant main or interaction effects were observed for negation. The results of selected measures of complexity, LCM and sentiment are presented in Fig 2, which shows a grid of raincloud plots based on the predicted values [27].

### Part-of-speech and over-generalizations

#### Pronouns

The results of our hierarchical regression indicate no differences between true and deceptive statements in the number of third or non-third person pronouns. The transcription condition had more non-third person pronouns than the written condition (*b* = 0.002, *p* = 0.001), and third-person pronouns were used more often in written than transcribed statements (*b* = -0.003, *p* = 0.001). No significant interaction effects were observed.

#### Infinitives

Our results indicate that the participants used a greater number of infinitives in the condition of false statements than in the condition of true statements (*b* = 0.004, *p* = 0.013). The main effect of statement form (written vs. transcribed) was not statistically significant.

No significant main effect of statement type (lie vs. truth) was found for any other part-of-speech variable (the complete list of analyzed variables can be found in the S1 File).

#### Over-generalisations

No significant main or interaction effects were observed for over-generalization.

### Model predictions

After checking the differences between true and false statements, the next step was to train and evaluate a model using the features we described above. In total, the feature space consisted of 43 variables covered in the former sections, including part-of-speech frequencies, LCM as individual variables and overall score, and text complexity scores. In addition, the features were extended with named entities, computed by the Spacy tool.

Table 2 contains the averaged accuracy of 20-fold cross-validation (along with the standard error provided after the ± mark). Two types of classification algorithms were applied: Support Vector Machine with the radial kernel implemented using the SVC API of Scikit-learn [28] and the XGBoost algorithm [29]. Both are considered state-of-the-art among non-deep learning methods. Overall, the performance of the SVM algorithm is better than that of XGBoost, with the best results achieved using written utterances. We did not fine-tune hyperparameters.

**Table 2:** Machine learning predictions: Averaged accuracy of 20-fold cross validation.

Utterances (number)	Model	Accuracy
All (1497)	SVM	0.576 ± 0.013
	XGBoost	0.566 ± 0.013
Transcribed (737)	SVM	0.597 ± 0.018
	XGBoost	0.580 ± 0.018
Written (760)	SVM	0.603 ± 0.018
	XGBoost	0.531 ± 0.018

## Discussion

Our research attempted to determine whether the linguistic differences found between true and false statements in previous studies conducted in English are also present in Polish. The biggest difference between English and Polish is the rich morphosyntax of the latter, as well as case, gender, and other properties encoded in word forms through the inflection system. These differences seemed likely to produce slightly different results than those described for the English language when differentiating between truth and lying.

We also wanted to replicate the results obtained by other researchers using selected newer and more precise methods to compute complexity than the LIWC category that has been used so far. Our methods allowed us to determine which aspects of language complexity are affected by the cognitive load associated with lying. We also decided to check whether written lies are different from spoken ones.

### Complexity

In our research, we used three different measures that may indicate complexity: two of them (MDD and MHD) relate only to the syntax of the utterance, while one focuses on word lengths and also accounts for sentence lengths (FOG). Differences between true and false statements were only visible in the FOG results, which means that our participants built equally complex statements when lying or telling the truth in terms of syntax. The greater cognitive load of lying was reflected in the vocabulary: when telling the truth, participants were able to use more complex and sophisticated vocabulary than when lying. This provides additional details concerning the results obtained by researchers in previous studies [10], as it actually seems that lying is more cognitively demanding but does not affect a statement’s syntax.

### Length

All true utterances obtained in our study were significantly longer than false ones. As in other studies [7, 30, 31] in which the true statements turned out to be longer than false statements, we believe it is related to the topic on which participants expressed their opinion. These were issues on which they had an established opinion, and they were therefore able to provide more arguments, facts, details, and finally their own beliefs.

In the case of written statements, the opposite results were obtained in a previous study [32], but the task in that study involved convincing a partner of a non-verifiable opinion in which the participants may have used additional discourse to provide reasons and arguments for their deceptions. In our research, participants focused rather on verifiable facts when writing. They were probably able to give more facts consistent with their true opinion.

### Concreteness

The LCM is an advanced way to measure the level of abstractness of a language. It takes into account all verbs, which it divides into three types in this respect, as well as selected adjectives, where the choice is dictated by the sentence syntax and the dependency parser.

Contrary to Newman et al. [10], our results show that participants did not use concrete verbs more often when making false statements. This difference is probably caused by a much broader approach to the category of concreteness within the LCM model than in Newman’s research, where only motion verbs were considered concrete. When assessing concreteness overall and taking into account all types of verbs and adjectives listed under the LCM, true statements were characterized by a higher level of abstraction than false statements. This is because, that lying is so cognitively demanding that it requires constructing simpler and less abstract statements [10].

### Sentiment and negations

We obtained very interesting results in terms of sentiment, showing that true statements were characterized by a greater number of negative terms, while deceitful statements included more positive ones. This result may be related to the method itself. Contrary to previous research, we used sentiment analysis rather than LIWC word categories. Sentiment analysis, as used in our work, follows the categories Positiv and Negativ as in the General Inquirer dictionary [33]. This, as well as the relatively large size of the Polish sentiment dictionary (the total size of the LIWC dictionary is 2300 words, whereas the sentiment dictionary used in our study exceeds 5400 words), enables the categorization of a much larger number of words, including more ambiguous ones with weaker polarization.

Another possible explanation implies that in the case of most topics, the true attitude of the participants was negative—they did not like something, did not support it, or did not consider it to be good. Therefore, in the case of true opinions, they used more negative terms. This is likely also the reason why we did not note any differences between true and false utterances in the case of negation words.

### Part-of-speech and over-generalisations

In Polish, the distinction between the speaker (first person), the addressee (second person), and others (third person) is not only a matter of personal pronouns that precede verbs (e.g. “I did”). Instead, it is contained in the morphosyntax of verbs and their conjugation (in Polish “zrobiłem”). In our view, this is the main reason why our participants did not use third- and non-third person pronouns more often while lying. We believe that this is typical of the Polish language, where personal pronouns are relatively rarely expressed directly in sentences. In previous studies, the less frequent use of first-person pronouns by liars was related to the desire to distance themselves from their lie [34]. We suggest that our participants used infinitives more often for the same purpose in Polish, as false statements contained a greater number of infinitives. The more frequent use of the impersonal form of a verb is characteristic of a less direct way of constructing statements.

We analyzed the frequency of many parts of speech in our study, but found differences between true and false statements in only two of them. It seems that focusing on parts of speech that enable the encoding of shallow syntactic information is insufficient and deep syntax should also be considered. In a study conducted by Feng et al. [35] in English, such differences between truth and falsehood were found using selected features driven by Probabilistic Context Free Grammar PCFG parse trees. We also found no difference in the use of over-generalization between true and false utterances. This result is consistent with that obtained by Vrij [5]. Finally, we found no differences between written and spoken lies. This may be due to the method of data collection and the fact that written and oral statements were produced by the same individuals in our study.

### Model predictions

The model we trained achieved similar results in classifying true and false statements as analogous models in the English language [10]. However, machine learning models using predefined lexicon-based features do not achieve state-of-the-art results in automatic deception detection. The best results are currently obtained by models based on deep, pre-trained transformer neural networks. To improve our results it would probably be useful to include audio-prosodic characteristics of statements. As Chen’s [36] research shows, including disfluencies and prosody significantly improves the statement recognition performance of automatic models.

### Limitations

We tried to use universal measures that can be applied regardless of what language the materials are in. However, this was not possible for all variables. For example, LCM is a dictionary method with different language versions, but it is still not available for all languages. Most importantly, our results concerning the morphology and parts of speech such as the lack of differences in the case of pronouns or many other parts of speech may be unique to Polish.

The second limitation is related to motivation. Our participants knew that other people would judge their statements and try to guess whether they are true or false, but this was the only manipulation used to increase their motivation to lie effectively. Unreliable lies have no consequences, and research shows that motivation can be an important moderator of which markers reveal deception [5].

A natural next step in our research would be to directly compare our chosen methods’ effectiveness with LIWC. Unfortunately, this is not possible at the moment because LIWC is not openly available in Polish.

### Conclusions

Using advanced tools, we verified the results of previous research on the detection of lying by textual analysis. We confirmed some of the conclusions drawn from them, but most of all we managed to refine and expand on results regarding the complexity of statements. In our research, it turned out that lying—being cognitively demanding—can actually affect a statement’s complexity level, but mainly its vocabulary rather than its syntax. False statements also turned out to be more concrete than true statements.

We believe this can be particularly useful in studies where the cognitive load of subjects is manipulated in order to detect lying more effectively. An interesting direction for further research would be to determine the effect of cognitive ability, such as executive functioning and memory, on differences in complexity between true and false statements. New computer textual analysis tools enable the precise measurement of many variables and can be used by researchers to analyze statements in languages other than English. They can be used both to clarify the current knowledge about linguistic deception indicators and to look for new features that distinguish true statements from false statements.

## Supporting information

S1 File List of variables—Parts-of-speech. (PDF)

S1 Table Means and standard deviations for all variables by truth/lie and written/transcribed condition. (PDF)

S2 Table Summary of models. (PDF)
