# Supplementary material for: Truth or lie: Exploring the language of deception
Source: PLoS One. 2023 Feb 2;18(2):e0281179. doi: 10.1371/journal.pone.0281179 (PMC9894434; doi:10.1371/journal.pone.0281179)
Supplement: S1 File — (PDF) [file pone.0281179.s001.pdf]

Part-of-speech:

- 3rd person pronoun
- non-third pronoun
- preposition
- substantive
- conjunction
- verb form not-past
- adjective
- [adjectival] present participle
- pseudo Participle
- adverb
- numeral
- comparative
- predicative
- iinfinitive
- [adjectival] past participle
- pronoun ‘siebie’
- winien; male singular form
- verb imperative
- predicative adjective
- agglutinate być
- interjection
- ad-adjectival adjective
- contemporary adv. participle
