# Supplementary material for: Truth or lie: Exploring the language of deception
Source: PLoS One. 2023 Feb 2;18(2):e0281179. doi: 10.1371/journal.pone.0281179 (PMC9894434; doi:10.1371/journal.pone.0281179)
Supplement: S1 Table — (PDF) [file pone.0281179.s002.pdf]

S1 Table. Means and standard deviations for crucial variables by truth/lie and written/transcribed conditions.

	TRUTH		LIE		WRITTEN		TRANSCRIBED	
	M	SD	M	SD	M	SD	M	SD
MHD	2,607	,614	2,523	,584	2,347	,614	2,790	,629
MDD	3,011	,513	2,946	,523	2,718	,513	3,247	,468
FOG	14,328	6,050	13,336	5,140	13,387	6,050	14,294	4,761
Sentence length	9,57	5,829	8,50	4,797	7,41	5,829	10,71	6,204
Characters	1395,44	973,672	1138,29	773,501	812,72	973,672	1735,71	1008,376
Tokens	246,74	179,689	201,20	141,409	315,09	179,689	315,09	182,221
LCM*	8,639	14,306	6,945	9,211	4,677	14,306	11,009	15,803
DAV*	,206	,192	,188	,145	,143	,192	,253	,199
IAV*	,222	,154	,204	,131	,181	,154	,246	,156
SV*	,351	,238	,330	,212	,253	,238	,431	,249
LCM_ADJ*	,008	,007	,008	,008	,008	,007	,009	,007
Non-third-person-pronoun*	,007	,008	,007	,009	,006	,008	,008	,008
Third-person-pronoun*	,012	,011	,014	,013	,015	,011	,011	,011
Infinitives*	,027	,019	,031	,020	.028	,019	,030	,017
Negations*	,025	,016	,026	,019	,024	,016	,027	,017
Generalizations*	,004	,006	,005	,008	,004	,006	,005	,007
Positive sentiment*	,050	,026	,055	,030	,058	,026	,047	,023
Negative_sentiment*	,028	,021	,023	,020	,030	,021	,020	,014

* Variables divided by the number of tokens.
