# Supplementary material for: Truth or lie: Exploring the language of deception
Source: PLoS One. 2023 Feb 2;18(2):e0281179. doi: 10.1371/journal.pone.0281179 (PMC9894434; doi:10.1371/journal.pone.0281179)
Supplement: S2 Table — (PDF) [file pone.0281179.s003.pdf]

S2 Table. Summary of regression models

	LCM*			positive sentiment*			negative sentiment*			MHD			MDD			FOG		
Predictors	*Estimates*	*CI*	*p*	*Estimates*	*CI*	*p*	*Estimates*	*CI*	*p*	*Estimates*	*CI*	*p*	*Estimates*	*CI*	*p*	*Estimates*	*CI*	*p*
**Intercept**	1.83	1.78 – 1.87	**<0.001**	0.05	0.05 – 0.05	**<0.001**	0.02	0.02 – 0.03	**<0.001**	1.26	1.25 – 1.27	**<0.001**	1.37	1.37 – 1.38	**<0.001**	2.64	2.62 – 2.66	**<0.001**
T_vs_L	-0.12	-0.18 – -0.06	**<0.001**	0.01	0.00 – 0.01	**<0.001**	-0.00	-0.01 – -0.00	**<0.001**	-0.02	-0.04 – -0.01	**<0.001**	-0.02	-0.03 – -0.01	**0.001**	-0.06	-0.09 – -0.03	**<0.001**
W_vs_T	0.62	0.56 – 0.68	**<0.001**	-0.01	-0.01 – -0.01	**<0.001**	-0.01	-0.01 – -0.01	**<0.001**	0.12	0.11 – 0.14	**<0.001**	0.13	0.12 – 0.14	**<0.001**	0.09	0.06 – 0.11	**<0.001**
T_vs_L * W_vs_T	-0.16	-0.27 – -0.04	**0.011**	0.00	-0.00 – 0.01	0.451	0.00	-0.00 – 0.01	0.112	-0.00	-0.03 – 0.02	0.716	-0.00	-0.02 – 0.02	0.873	-0.02	-0.07 – 0.04	0.539
**Random Effects**																		
σ^2	0.35			0.00			0.00			0.02			0.01			0.07		
τ_{00}	0.13			0.00			0.00			0.01			0.00			0.02		
ICC	0.27			0.14			0.13			0.26			0.21			0.23		
N	415			415			415			415			415			415		
Observations	1497			1497			1497			1497			1497			1497		
Marginal R^2 / Conditional R^2	0.176 / 0.398			0.047 / 0.182			0.072 / 0.191			0.148 / 0.369			0.272 / 0.424			0.028 / 0.256		

T-truth, L-lie, W-written, T-transcribed. * variables scaled (divided by the number of tokens)
